# The Double-Edged Sword Effects of Maternal Nutrition in the Developmental Programming of Hypertension

**DOI:** 10.3390/nu10121917

**Published:** 2018-12-04

**Authors:** Chien-Ning Hsu, You-Lin Tain

**Affiliations:** 1 Department of Pharmacy, Kaohsiung Chang Gung Memorial Hospital, Kaohsiung 833, Taiwan; chien_ning_hsu@hotmail.com; 2 School of Pharmacy, Kaohsiung Medical University, Kaohsiung 807, Taiwan; 3 Department of Pediatrics, Kaohsiung Chang Gung Memorial Hospital and Chang Gung University College of Medicine, Kaohsiung 833, Taiwan; 4 Institute for Translational Research in Biomedicine, Kaohsiung Chang Gung Memorial Hospital and Chang Gung University College of Medicine, Kaohsiung 833, Taiwan

**Keywords:** developmental programming, fat, fructose, hypertension, nutrition, pregnancy, reprogramming

## Abstract

Hypertension is a growing global epidemic. Developmental programming resulting in hypertension can begin in early life. Maternal nutrition status has important implications as a double-edged sword in the developmental programming of hypertension. Imbalanced maternal nutrition causes offspring’s hypertension, while specific nutritional interventions during pregnancy and lactation may serve as reprogramming strategies to reverse programming processes and prevent the development of hypertension. In this review, we first summarize the human and animal data supporting the link between maternal nutrition and developmental programming of hypertension. This review also presents common mechanisms underlying nutritional programming-induced hypertension. This will be followed by studies documenting nutritional interventions as reprogramming strategies to protect against hypertension from developmental origins. The identification of ideal nutritional interventions for the prevention of hypertension development that begins early in life will have a lifelong impact, with profound savings in the global burden of hypertension.

## 1. Introduction

Hypertension remains an important public health challenge, despite treatment advances over the past decades. However, hypertension is a disease of multifactorial origins that can be treated to prevent more related disorders if found early. Although hypertension is more common in adults, it can occur at any age. Indeed, adult-onset hypertension can originate in early life [1]. Maternal nutrition during pregnancy and lactation has important implications for optimal fetal development and long-term health of the offspring. Imbalanced maternal nutrition produces fetal programming that permanently alters the body’s morphology and function and leads to many adult diseases, including hypertension [2]. This notion is framed as the developmental origins of health and disease (DOHaD) [3]. Conversely, the DOHaD concept leads to a shift in the therapeutic approach from adult life to early stage, before hypertension is evident. This strategy reversing the programming processes in fetal and infantile life is known as reprogramming [4]. Nutrition interventions have recently started to gain importance as a reprogramming strategy to prevent hypertension of developmental origins [4,5,6].

**Figure 1:**
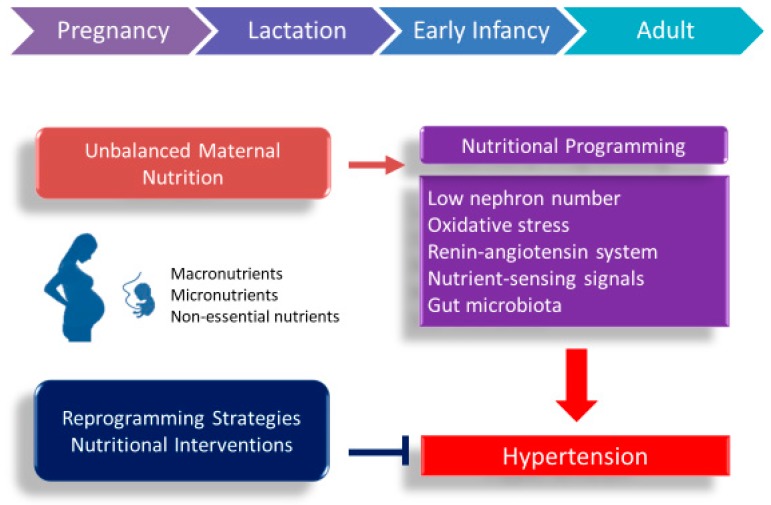
Schematic illustration of the double-edged sword effects of maternal nutrition and common mechanisms underlying the developmental programming of hypertension.

According to the two aspects of the DOHaD concept, maternal nutrition may play an important role as a double-edged sword in developmental programming of hypertension. This review will first present the clinical and experimental evidence for how maternal undernutrition and overnutrition trigger the programming mechanisms leading to programmed hypertension. This will be followed by potential nutritional interventions that may serve as a reprogramming strategy to halt the growing epidemic of hypertension. A schematic summarizing the links between maternal nutrition, early-life insults, and mechanisms underlying programming of hypertension is presented in Figure 1.

## 2. Evidence for Programming of Hypertension Related to Maternal Nutrition Status in Humans

Epidemiologic studies support that malnutrition during gestation and lactation has lifelong consequences on adult offspring’s health. A well-known example comes from the Dutch Hunger Winter Families study [7]. Offspring exposed to maternal famine develop many adult diseases, including hypertension [7,8,9]. Another line of evidence for reinforcement comes from observations of malnutrition leading to preterm birth. Epidemiologic studies now support that preterm birth is a key risk factor for hypertension in later life [10]. A meta-analysis of 10 studies with 1342 preterm participants shows that preterm birth subjects have modestly higher systolic blood pressure (BP) later in life [11]. Besides, numerous reports indicate that intakes or lack of specific nutrients during pregnancy may increase the risk of preterm birth [12]. Additionally, the risk of programmed hypertension has been examined in mother–child cohorts (Table 1). Several nutritional risks correlated with the elevation of BP in offspring in these cohorts include vitamin D deficiency [13,14], short-term breastfeeding [15], gestational diabetes mellitus [16], excessive gestational weight gain [17,18], macronutrient intake deficiency [19], and undernutrition [9]. It is noteworthy that hypertension can develop in early childhood [13,15,16], but tends to occur in adulthood. All of these observations provide a link between the imbalanced maternal nutrition status and the risk of developing hypertension in later life.

Nevertheless, these cohorts cannot per se directly establish a causal relationship between the maternal nutrition status and hypertension phenotype in offspring. Also, these cohorts do not illuminate molecular mechanisms by which the hypertension phenotype is created. As a consequence of ethical considerations concerning what is feasible or not in human studies, animal models are of great importance. It is obvious that much of our knowledge comes from animal models, which identify that specific types of nutrients may program hypertension phenotypes. The developmental window is critical for nutritional programming, and specific nutrition interventions can be used as reprogramming strategies.

## 3. Animal Models of Programmed Hypertension Induced by Imbalanced Maternal Nutrition 

Excessive or insufficient consumption of a specific nutrient has been linked to developmental programming of hypertension. Here, we mainly summarize some of the rodent studies documenting BP phenotypes in offspring after a variety of nutritional interventions (Table 2) [20,21,22,23,24,25,26,27,28,29,30,31,32,33,34,35,36,37,38,39,40,41,42,43,44,45,46,47]. We have restricted this review to nutritional interventions ending before the start of weaning. As shown in Table 2, rats are the most commonly used subjects among the small animal models. Rats reach sexual maturity at approximately 5–6 weeks of age. In adulthood, one rat month is roughly equivalent to three human years [48]. Accordingly, Table 2 lists the timing of developing hypertension measured in rodents with different ages, which can be calculated and translated to humans of a specific age group. It concerns both undernutrition [20,21,22,23,24,25,26,27,28,42,43,44,45,46,47] and overnutrition [30,31,32,33,34,35,36,37,38,39,40]. It is noteworthy that many studies focused only on the male offspring [22,25,26,29,30,31,34,35,38,39,40,41,47].

Dietary nutrients can be divided to macronutrients, micronutrients, and non-essential nutrients. Macronutrients, which provide energy, include carbohydrates, proteins, and fats. Restriction of calories to various degrees (ranging from 30% to 70%) in pregnant dams has been reported to cause hypertension in their adult offspring [20,21,22,23]. Offspring exposed to a more severe degree of caloric restriction are likely to develop hypertension earlier. Rodent models of low protein feeding have been extensively used to study the mechanisms of nutritional programming. Similar to caloric restriction, a greater degree of protein restriction causes an earlier development of hypertension in offspring [24,25,26,27,28]. However, a previous study showed that two divergent low-protein (9%) diet manipulations in rat pregnancy provoked different programming effects on the offspring’s BP [49]. Of note, the balance of protein and other nutrients may be a critical determinant of the long-term effects of maternal low-protein diet on programming of hypertension. Pregnant women are now recommended to eat methyl donor food to reduce adverse birth outcomes [50]. Such methyl donor nutrients include methionine, choline, folic acid, and vitamins B2, B6, and B12. However, we recently found that pregnant rats fed with high methyl-donor diet or methyl-deficient diet resulted in programmed hypertension in their male adult offspring [29]. 

Fat is another macronutrient. A high-fat diet is a commonly used animal model to induce obesity and related disorders, like hypertension [51]. The observations of maternal high-fat diet-induced hypertension in offspring are varied [52]. Maternal high-fat intake induced responses of BP include an increase [30,31,32] or no change [31,33], mainly depending on sex, age, strain, and diverse fatty acids compositions. Additionally, several studies showed that consumption of high-fructose alone or as a part of diet by rodent mothers induces programmed hypertension in adult offspring [34,35,36,37,38,39]. Fructose is a monosaccharide naturally present in fruits and honey. However, most of the increase in fructose consumption now is derived from high fructose corn syrup and table sugars. A previous report revealed that up to 74% of fructose came from processed foods and beverages other than whole fruits and vegetables [53]. A maternal high-fructose diet is being developed into a commonly used animal model to induce metabolic syndrome of developmental origins [54]. Although most studies have used fructose doses amounting to ~60% of the total energy requirement [36,37,38], evidence indicates that maternal consumption of 10% w/v fructose significantly increases BP in mice offspring after 1 year [39]. On the other hand, several studies used fructose as a part of maternal diet along with fat and salt [35,38,40]. Given that the Western diet is characterized by the intake of high-sugar drinks, high-fat products, and excess salt, it is important to elucidate the interplay between fructose, fat, and salt on the programming of hypertension. Indeed, animal studies examining the combined effects of key components of the Western diet have shown their synergistic effects between fructose, fat, and salt on the elevation of BP in adult offspring [38,55,56].

Besides, sodium, potassium, calcium, magnesium, and other ions are listed with macronutrients as they are required in large quantities. Interestingly, both low- and high-salt diet exposure during pregnancy and lactation have been reported to cause elevated BP in male adult offspring [41]. Maternal calcium-deficient diet increased BP in adult offspring [42], while magnesium-deficient diet did not [43].

Additionally, deficiencies in micronutrients, including trace elements and vitamins, in pregnant mothers are associated with the development of hypertension in their adult offspring [44,45,46,47]. These micronutrients include iron [44,45], vitamin D [46], and zinc [47]. Although vitamin C, E, B6, flavonoids, and coenzyme Q-10 have been shown to lower BP [57], whether deficiencies of these micronutrients on pregnant mothers leading to programmed hypertension in their offspring is still largely unknown. Besides, no studies have been conducted examining the role of deficient non-essential nutrients on programming of hypertension. In the current review, limited information is available about the use of large animals to study nutritional programming induced offspring hypertension. Two reports in cows and sheep showed that maternal undernutrition causes elevation of BP in adult offspring [58,59].

## 4. Common Mechanisms Underlie Nutritional Programming of Hypertension

Since various nutritional manipulations in gestation and lactation generate very similar outcomes with respect to hypertension in adult offspring in different species, these observations suggest the existence of common mechanisms that may contribute to the pathogenesis of hypertension of developmental origin. So far, programming of hypertension has been attributed to several mechanisms [4,60,61,62,63,64]. Some of these mechanisms that have been previously linked to nutritional programming include low nephron number, oxidative stress, activation of the renin–angiotensin system (RAS), nutrient-sensing signals, gut microbiota, and sex differences. Each will be discussed in turn.

### 4.1. Low Nephron Number

The kidneys are known to play a decisive role in regulation of BP. There is an increasing body of literature demonstrating the relationship between low nephron number and hypertension, as reviewed elsewhere [65,66]. Renal development in rodents, unlike in humans, continues up to postnatal week 1–2. Therefore, nutritional insults during last third of pregnancy and early lactation periods have been reported to impair nephrogenesis, leading to a reduction in nephron number [61]. As we reviewed elsewhere [67], these nutritional factors include caloric restriction [22], low-protein diet [25,68], high-salt diet [41], low-salt diet [41], vitamin A deficiency [69], and iron deficiency [70]. Maternal protein restriction resulted in reduced nephron number and elevation of BP in adult male offspring, which is related to renal hyperfiltration and activation of the renin–angiotensin system (RAS) [25]. Additionally, maternal iron and zinc deficiencies have also been found to reduce nephron number and increase systolic BP in adult offspring [66]. Conversely, nephron endowment can be unaltered [71], or even increased in response to nutritional programming [72]. These findings suggest that the nutritional programming of hypertension might be not specific to a single factor (i.e., low nephron endowment) and other mechanisms demands further exploration.

### 4.2. Oxidative Stress

Oxidative stress is an oxidative shift characterized by an imbalance between pro-oxidant molecules and antioxidant defenses. Nitric oxide (NO), a vasodilator and a free radical, is involved in BP control and oxidative stress. NO plays an important role in placental and fetal growth [73]. Early-life redox imbalance may lead to lifelong effects in vulnerable organs leading to hypertension in later life [74]. Offspring born to dams with NO deficiency develop hypertension [75], while restoration of the balance between NO and reactive oxygen species (ROS) was considered as a reprogramming strategy to prevent hypertension of developmental origins [76]. Numerous nutritional interventions have been reported to result in programmed hypertension related to oxidative stress, including caloric restriction [21,22], low protein diet [26], methyl-donor diet [29] high fat intake [30,32,33], high-fructose diet [36], and zinc deficiency diet [47]. Although oxidative stress is unlikely to be attributed to the sole mechanism that increases the vulnerability to later hypertension, it is required to elucidate its interplay with other mechanisms of nutritional programming in determining its impact on hypertension.

### 4.3. Renin–Angiotensin System

RAS is broadly involved in control of BP and pharmacological blockade of the RAS has been clinically used to treat hypertension. Inactivation of RAS components cause the reduction of nephron number [77]. Several nutritional insults during early life leading to predisposition toward dysregulation of RAS have been reported, including low-protein diet [25,28], high-fat diet [33], high-sucrose diet [34], and high-fructose diet [37]. Conversely, early blockade of the RAS has been reported to reprogram inappropriate activation of the RAS to prevent the developmental programming of hypertension [37,78,79]. These observations support the notion that RAS might be a common mechanism that underlies hypertension of developmental origins. It is noteworthy that blockade of the RAS to prevent the programmed hypertension cannot start as early as two weeks after birth in the rodent models for two reasons. First, angiotensin-converting enzyme inhibitors and angiotensin receptor blockers are contraindicated during pregnancy because of their teratogenic effects. Second, blockade of the RAS impairs nephrogenesis and nephrogenesis completes in postnatal weeks 1–2 in rodents. 

### 4.4. Nutrient-Sensing Signals

Nutrient-sensing signaling pathways influence fetal metabolism and development according to maternal nutritional status. Cyclic adenosine monophosphate (AMP)-activated protein kinase (AMPK) and peroxisome proliferator-activated receptors (PPARs) are well-known nutrient-sensing signals [80]. The interplay between AMPK and other nutrient-sensing signals, driven by maternal nutritional interventions were found to regulate PPARs and their target genes, thereby generating programming of hypertension [81]. Several genes involved in oxidative stress are PPAR target genes, such as *Nos2*, *Nos3*, *Sod2*, and *Nrf2* [82]. Additionally, PPARγ has been reported to stimulate renin gene expression [83]. Given that the RAS cascade starts with the release of renin from the kidney, it is possible that programmed hypertension is attributed to these PPAR target genes induced oxidative stress and RAS activation. Furthermore, PPARγ can increase several sodium transporters to increase sodium reabsorption, leading to programmed hypertension [84]. Therefore, nutritional insults could affect PPARs and their target genes to induce renal programming leading to programmed hypertension.

Conversely, pharmacological interventions targeting AMPK signaling has been considered as reprogramming strategies to prevent programmed hypertension [85]. Detailed mechanisms that underlie the interactions between maternal nutrition and nutrient-sensing signals and their roles in the programming process toward hypertension of developmental origins, however, remain unclear.

### 4.5. Gut Microbiota

Gut is the first organ in contact with dietary nutrients. Maternal nutritional insults may cause a microbial imbalance, namely dysbiosis [86]. Dysbiosis in early life has negative effects and may have long-term consequences leading to many adult diseases, including hypertension [86]. Emerging evidence shows that the development of hypertension is correlated with gut microbiota dysbiosis in animal models of hypertension [87,88,89]. Several possible mechanisms have been identified linking the dysbiosis and hypertension, including alterations of microbial metabolite short-chain fatty acids and their receptors, increases of microbiota-derived metabolite trimethylamine-N-oxide, increased sympathetic activity, activation of the RAS, and inhibition of NO as well as hydrogen sulfide [89]. In addition to macro- and micro-nutrients, non-essential nutrients are substances within foods that can have a significant impact on health, for example, dietary fiber. It is noteworthy that consumption of dietary fiber has become one dietary strategy for modulating the microbiota. Our recent report showed that modulation of gut microbiota by prebiotics (i.e., a special form of dietary fiber) or probiotics (i.e., beneficial bacteria in the gut) can prevent maternal high-fructose consumption induced programmed hypertension [90]. Despite recent studies demonstrating that microbiota-targeted therapies can be applied to a variety of diseases [91], their roles on programmed hypertension remain to be identified. 

### 4.6. Sex Differences

There now is a substantial literature indicating that sex differences exist in the developmental programming of hypertension [92,93], showing that males are more prone to hypertension than females. Besides, several above-mentioned mechanisms of renal programming, such as oxidative stress [94] and inappropriate activation of the RAS [95] have been reported to respond to early-life insults in a sex-specific manner. The long-term effects of the same nutritional insult, such as maternal caloric restriction [23], high-fat diet [33], or high-fructose diet [37], can induce various phenotypes on male and female offspring. This difference has led many researchers to target their efforts entirely to one sex, especially to males [22,25,26,29,30,31,34,35,38,39,40,41,47]. Nevertheless, only few studies have investigated the programming response to maternal diet focused on transcriptome profiles of the offspring kidney [96]. In a maternal high-fructose diet model [37], we found renal transcriptome is sex-specific and female offspring are more fructose-sensitive. This is in accord with a report revealing that more genes in the placenta were affected in females than in males under different maternal diets [97]. A previous study has shown that sex-specific placental adaptations are often associated with male offspring developing adult disease while females are minimally affected [98]. However, whether higher sensitivity to nutritional insults is beneficial or harmful for programming effects in female offspring awaits further evaluation.

## 5. Nutritional Interventions as Reprogramming Strategies to Prevent Programmed Hypertension

Reprogramming strategies to reverse the programming processes that have been employed include nutritional intervention, exercise and lifestyle modification, and pharmacological therapy [4,6]. Nutritional programming is hypothetically bidirectional: supplementing deleterious nutrients or depleting beneficial nutrients in pregnancy and lactation can induce hypertension, whereas programmed hypertension can be mitigated by maternal supplementation of beneficial nutrients. Although various clinical practice guidelines have been established to provide appropriate therapies for hypertensive disorders of pregnancy [99], less attention has been paid to focus on nutritional interventions. On the other hand, supplementation with macro- and micro-nutrients during pregnancy and lactation periods has been recommended to improve maternal and birth outcomes [100,101]. However, little is known whether supplementing with specific nutrition in early life can be beneficial on programmed hypertension induced by diverse early-life insults in humans. Thus, this review will restrict to nutritional interventions as reprogramming strategies to prevent hypertension of developmental origins in a variety of animal models, some of which are listed in Table 3 [21,22,75,90,102,103,104,105,106,107]. This list is by no means complete and is expected to grow rapidly as nutritional interventions recently started to gain importance in the field of DOHaD research [108].

Reprogramming strategies can be created based on the above-mentioned mechanisms leading to programmed hypertension. The reduced nephron number was restored by citrulline supplementation in the caloric restriction model [22]. The beneficial effects of citrulline [22,75], micronutrients [21], conjugated linoleic acid [106], and folic acid [107] on hypertension are related to reduction of oxidative stress. On the other hand, branched-chain amino acid supplementation prevented hypertension related to regulation of the RAS in the caloric restriction model [104]. Furthermore, maternal administration of inulin prevented adult rat offspring against high-fructose diet-induced programmed hypertension associated with nutrient-sensing signals [90].

Most reprogramming strategies have been directed at amino acids, including glycine [109], citrulline [22,75,102,103], branched-chain amino acid [104], and taurine [105]. Amino acids are building blocks of proteins and, hence, play crucial roles in organogenesis and fetal development. Glycine and vitamins (folic acid, vitamin B2, B6 and B12) take part in one-carbon metabolism and DNA methylation. Thus, glycine supplementation may have important implications for fetal programming through epigenetic mechanisms. Although a methyl-donor diet could be used for prevention of various human diseases [110], our recent study demonstrated that a maternal methyl-donor diet causes programmed hypertension in adult offspring [29]. Next, citrulline supplementation has been documented to be protective on adult offspring against hypertension in different models, including maternal caloric restriction [22], maternal NO deficiency [75], streptozotocin-induced diabetes [89], and prenatal dexamethasone exposure [103]. Citrulline supplementation is proposed to increase de novo synthesis of arginine (the substrate for nitric oxide synthase) and prevent NO deficiency [111]. Although postnatal arginine supplementation has been reported to prevent the development of hypertension in intrauterine restricted rats [112], the reprogramming effects of maternal arginine supplementation have not been examined in various models of programmed hypertension. Given that citrulline is mainly taken up by the kidney to generate arginine and that citrulline can also prevent some of the untoward effects of arginine supplementation, a better understanding of maternal citrulline supplementation in the prevention of programmed hypertension in other animal models is warranted before it is implemented in humans. Additionally, maternal branched-chain amino acid supplementation prevents developmental hypertension in adult rat offspring [104]. However, a previous report indicated that the dietary amino acid pattern, rich in branched chain amino acids, could increase the risk of hypertension [113]. Moreover, maternal taurine supplementation prevented maternal diabetes-induced programmed hypertension [105]. Taurine is an abundant semi-essential, sulfur-containing amino acid. It is well known to lower BP and increase hydrogen sulfide in established hypertensive models [114]. Since current evidence supports hydrogen sulfide as a reprogramming strategy for long-term protection against hypertension [115], whether the protective effects of maternal taurine supplementation on programmed hypertension is related to hydrogen sulfide pathway deserve further elucidation in other programming models. In addition to amino acids among macronutrients, only one report showed maternal conjugated linoleic acid supplementation has reprograming effects against hypertension [106]. Although long chain polyunsaturated fatty acids have been recommended for pregnant and breastfeeding women [101], their effects on programed hypertension remain to be determined. Next, two reports demonstrated reprogramming effects of micronutrients on programmed hypertension [21,107]. These micronutrients contain vitamin C, E, selenium, and folic acid. Vitamin C, E, and selenium have antioxidant properties. Folic acid is involved in DNA methylation. These micronutrients were shown to prevent programmed hypertension by restoring NO and reducing oxidative stress [21,107]. Furthermore, non-essential nutrients could be reprogramming strategies to prevent programmed hypertension. Maternal supplementation with dietary fiber was reported to prevent programmed hypertension in adult offspring born to dams fed with high-fructose diet [89]. Although combined supplementations with high-fiber diet and short-chain fatty acid acetate prevent hypertension in deoxycorticosterone acetate (DOCA)–salt hypertensive mice [116], their reprogramming effects on models of developmental programming awaits further elucidation. Considering that the gut microbiota dysbiosis has been associated with hypertension, supplementation with prebiotics or other nutritional interventions targeting the microbiota could be used as a reprogramming strategy. 

Moreover, growing evidence from animal studies suggests that sex differences in the kidney could be a key factor in the developmental programming of hypertension [92,93]. Further work is warranted to recognize the influences of sex differences on programmed hypertension that will aid in developing novel sex-specific strategies to prevent hypertension of developmental origin in both sexes.

## 6. Conclusions

Maternal nutrition is like a double-edged sword. A growing body of evidence suggest that supplementation with specific macro- and micro-nutrients, and even non-essential nutrients in pregnancy and lactation protect adult offspring against hypertension in a variety of models of developmental origins. Yet, at the same time, it needs to be aware that unbalanced maternal nutrition not only affects maternal health, it also has an impact on fetal programming leading to programmed hypertension. Nutritional intervention as a reprogramming strategy against the development of hypertension is a great opportunity and will become even more crucial with the growing epidemic of hypertension and related disorders. These strategies have been proven effective in animal models, as shown earlier. A better understanding of mechanisms underlying nutritional programming is urgently needed before these interventions are implemented in humans. 

## Figures and Tables

**Table 1:** Effects of maternal nutrition on offspring blood pressure in human cohort studies.

Cohort Study	Offspring, *n*	Age Range, Year	Country	Risk Factors
ABCD [13]	1834	5–6	Netherlands	Vitamin D deficiency
Tohoku Study of Child Development [15]	377	7	Japan	Short-term breastfeeding
Hyperglycemia and Adverse Pregnancy Outcome study [16]	970	7	Hong Kong	Gestational diabetes mellitus
ALSPAC [14]	3525	9.9	United Kingdom	Vitamin D deficiency
ALSPAC [17]	2200	16	United Kingdom	Excessive gestational weight gain
DaFO88 [19]	434	20	Scotland	Macronutrient intake deficiency
MUSP [18]	2271	21	Australia	Excessive gestational weight gain
Dutch Famine study [9]	359	59	Netherlands	Undernutrition

Studies tabulated according to offspring age. ABCD = Amsterdam Born Children and their Development; ALSPAC = The Avon Longitudinal Study of Parents and Children; DaFO88 = Danish Fetal Origins Cohort; MUSP = Mater-University Study of Pregnancy and its Outcomes.

**Table 2:** Offspring blood pressure in nutritional rodent models of developmental programming.

Animal Models	Intervention Period	Species/Gender	Age at Measure (Week)	Higher than Control	Reference
Macronutrients					
30% caloric restriction	Pregnancy	Wistar/M+F	54	Yes	[20]
50% caloric restriction	Pregnancy	Wistar/M+F	14–16	Yes	[21]
50% caloric restriction	Pregnancy and lactation	SD/M	12	Yes	[22]
70% caloric restriction	Gestation days 0–18	Wistar/M+F	28	Yes	[23]
Protein restriction, 6%	Pregnancy	SD/F	52	Yes	[24]
Protein restriction, 8.5%	Pregnancy	SD/M	20	Yes	[25]
Protein restriction, 9%	Pregnancy	Wistar/M	12	Yes	[26]
Protein restriction, 9%	Pregnancy	Wistar/M+F	22	Yes	[27]
Protein restriction, 9%	1 week before conception and throughout pregnancy	FVB/NJ mice/F	24	Yes	[28]
High methyl-donor diet	Pregnancy and lactation	SD/M	12	Yes	[29]
Methyl-deficient diet	Pregnancy and lactation	SD/M	12	Yes	[29]
High-fat diet, 24%	Lactation	Wistar/M	22	Yes	[30]
High-fat diet, 25.7%	Lactation	SD/M	25	No	[31]
High-fat diet, 25.7%	Lactation	SD/F	25	Yes	[31]
High-fat diet, 45%	Pregnancy and lactation	C57BL6J mice/M	30	Yes	[32]
High-fat diet, 58%	5 weeks before the delivery and throughout pregnancy and lactation	SD/M+F	25	No	[33]
20% *w*/*v* sucrose in drinking water	Pregnancy	SD/M	90	Yes	[34]
10% *w*/*v* fructose plus 4% NaCl in drinking water	4 weeks before conception and throughout pregnancy and lactation	SD/M	9	Yes	[35]
High-fructose diet, 60%	Pregnancy and lactation	SD/M+F	12	Yes	[36,37]
High-fructose diet, 56.7% plus high-fat diet	Pregnancy and lactation	SD/M	16	Yes	[38]
10% *w*/*v* fructose in drinking water	Pregnancy and lactation	C57BL6J mice/M	52	Yes	[39]
High-fat diet, 45% plus 4% NaCl in drinking water	3 weeks before conception and throughout pregnancy and lactation	SD/M	19	Yes	[40]
Low-salt diet, 0.07%	Pregnancy and lactation	SD/M	21	Yes	[41]
High-salt diet, 3%	Pregnancy and lactation	SD/M	21	Yes	[41]
Calcium-deficient diet	Pregnancy	WKY/M+F	52	Yes	[42]
Magnesium-deficient diet	Pregnancy	C57BL6J mice /M+F	24	No	[43]
Micronutrients					
Iron restriction	4 weeks before conception and throughout pregnancy	RHL/M+F	10	Yes	[44]
Iron restriction	4 weeks before conception and throughout pregnancy	Wistar/M+F	64	Yes	[45]
Vitamin D restricted diet	6 weeks before conception and throughout pregnancy and lactation	SD/M+F	7–8	Yes	[46]
Zinc-deficient diet	Pregnancy and lactation	Wistar/M	12	Yes	[47]

Studies tabulated according to nutritional intervention and age at measure. SD = Sprague-Dawley rat; WKY = Wistar-Kyoto rat; M = Male; F = Female; RHL = Rowett Hooded Lister rat.

**Table 3:** Reprogramming strategies aimed at nutritional interventions to prevent hypertension of developmental programming in animal models.

Nutritional Interventions	Animal Models	Intervention Period	Species/Gender	Age at Measure (Week)	Lower BP?	Ref.
Macronutrients						
Glycine	Maternal 9% protein restriction	Pregnancy	Wistar/M	4	Yes	[109]
Citrulline	Maternal 50% caloric restriction	Pregnancy and lactation	SD/M	12	Yes	[22]
Citrulline	Maternal nitric oxide deficiency	Pregnancy and lactation	SD/M	12	Yes	[75]
Citrulline	Streptozotocin-induced diabetes	Pregnancy and lactation	SD/M	12	Yes	[102]
Citrulline	Prenatal dexamethasone exposure	Pregnancy and lactation	SD/M	12	Yes	[103]
Branched-chain amino acid	Maternal 70% caloric restriction	Pregnancy	SD/M	16	Yes	[104]
Taurine	Streptozotocin-induced diabetes	Pregnancy and lactation	Wistar/M+F	16	Yes	[105]
Conjugated linoleic acid	Maternal high-fat diet	Pregnancy and lactation	SD/M	18	Yes	[106]
Micronutrients						
Micronutrients: vitamin C, E, selenium and folic acid	Maternal 50% caloric restriction	Pregnancy	Wistar/M+F	14–16	Yes	[21]
Folic acid	Protein restriction, 9%	Pregnancy	Wistar/M	15	Yes	[107]
Non-essential nutrients						
Long chain inulin	Maternal high-fructose diet	Pregnancy and Lactation	SD/M	12	Yes	[90]

Studies tabulated according to types of nutritional intervention and age at measure. SD = Sprague–Dawley rat. M = male. F = female.
